# Bis{1,2-bis­[bis­(3-meth­oxy­prop­yl)phosphan­yl]ethane-κ^2^ *P*,*P*′}dichlorido­osmium(II)

**DOI:** 10.1107/S1600536811048926

**Published:** 2011-11-23

**Authors:** Nathaniel K. Szymczak, Lev N. Zakharov, David R. Tyler

**Affiliations:** a Department of Chemistry, 1253 University of Oregon, Eugene, Oregon 97403-1253, USA

## Abstract

In the centrosymmetric title compound, [OsCl_2_(C_18_H_40_O_4_P_2_)_2_], the Os^II^ atom adopts a *trans*-OsCl_2_P_4_ geometry, arising from its coordination by two chelating diphosphane ligands and two chloride ions. One of the meth­oxy side chains of the ligand is disordered over two orientations in a 0.700 (6):0.300 (6) ratio.

## Related literature

For background to transition-metal dihydride complexes, see: Egbert *et al.* (2007 ▶); Heinekey *et al.* (2004 ▶); Miller *et al.* (2002 ▶); Szymczak & Tyler (2007 ▶); Szymczak *et al.* (2006 ▶).
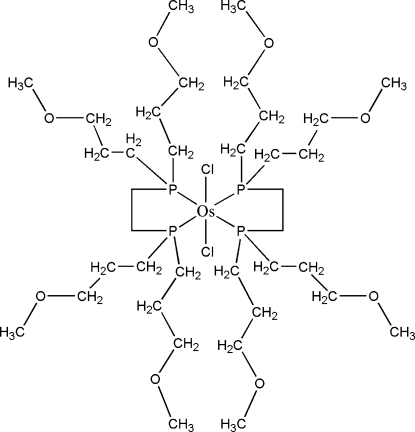


## Experimental

### 

#### Crystal data

[OsCl_2_(C_18_H_40_O_4_P_2_)_2_]
*M* *_r_* = 1025.98
Monoclinic, 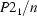

*a* = 12.667 (3) Å
*b* = 10.321 (2) Å
*c* = 18.754 (4) Å
β = 107.779 (3)°
*V* = 2335.0 (9) Å^3^
*Z* = 2
Mo *K*α radiation
μ = 3.03 mm^−1^
*T* = 173 K
0.14 × 0.10 × 0.04 mm

#### Data collection

Bruker APEX CCD diffractometer
Absorption correction: multi-scan (*SADABS*; Sheldrick, 1995 ▶) *T*_min_ = 0.677, *T*_max_ = 0.889
25950 measured reflections
5333 independent reflections
4423 reflections with *I* > 2σ(*I*)
*R*_int_ = 0.032

#### Refinement

*R*[*F* ^2^ > 2σ(*F* ^2^)] = 0.024
*wR*(*F* ^2^) = 0.058
*S* = 1.04
5333 reflections
251 parameters
5 restraints
H-atom parameters constrained
Δρ_max_ = 0.87 e Å^−3^
Δρ_min_ = −0.51 e Å^−3^

### 

Data collection: *SMART* (Bruker, 2000 ▶); cell refinement: *SAINT* (Bruker, 2000 ▶); data reduction: *SAINT* (Bruker, 2000 ▶); program(s) used to solve structure: *SHELXTL* (Sheldrick, 2008) ▶; program(s) used to refine structure: *SHELXTL* (Sheldrick, 2008) ▶; molecular graphics: *SHELXTL* (Sheldrick, 2008) ▶; software used to prepare material for publication: *SHELXTL* (Sheldrick, 2008) ▶.

## Supplementary Material

Crystal structure: contains datablock(s) I, global. DOI: 10.1107/S1600536811048926/hb6479sup1.cif

Structure factors: contains datablock(s) I. DOI: 10.1107/S1600536811048926/hb6479Isup2.hkl

Additional supplementary materials:  crystallographic information; 3D view; checkCIF report

## Figures and Tables

**Table 1:** Selected bond lengths (Å)

Os1—P2	2.3383 (8)
Os1—P1	2.3434 (8)
Os1—Cl1	2.4515 (8)

**Fig. 1.**
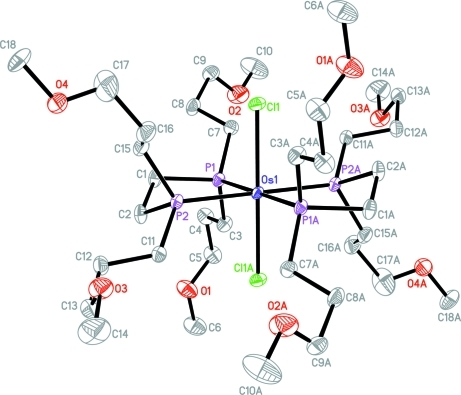
The structure of (I) with 50% probability displacement ellipsoids [Symmetry code (A): -x,-y,-z]. Only one position of the disordered C17/O4/C18 group is shown for clarity.

## supplementary crystallographic information

### Comment

A consequence of the large d-orbitals in third-row transition metals is increased overlap to antibonding orbitals of ligands such as CO and H_2_. Accordingly, when most third-row transition metal complexes are reacted with H_2_, the product is often a dihydride complex (Egbert et al., 2007; Szymczak & Tyler, 2007). However, when the d-orbitals are not sufficiently electron rich to promote a complete oxidative-addition product, an arrested oxidative addition can result.  The "arrested" intermediates belong to an unusual class of dihydrogen complexes with a bond distance between 1.1 Å and 1.5 Å (Heinekey et al., 2004).  The H-H bond in these complexes sits in an unusually flat potential energy surface, and the H-H bond distance is consequently very sensitive to changes in the environment around the coordinated η^2^-H_2_ ligand. We hypothesized that small changes to the environment around the H_2_ ligand could potentially be correlated with small energetic fluctuations, such as those that are typical of weak intermolecular forces such as hydrogen bonding.

Several osmium dihydrogen complexes have previously been shown to have H-H bond distances in the elongated regime (1.1 - 1.5 Å). For our study of hydrogen bonding of water to the H_2_ ligand, we sought therefore to study hydrogen bonding to the H_2_ ligand in the water-soluble trans-Os(DMeOPrPE)_2_(H_2_)H^+^ complex. (DMeOPrPE is the water-soluble, bidentate phosphine ligand 1,2-bis(bis(methoxypropyl)phosphanyl)ethane.)  In prior work, we synthesized trans-Fe(DMeOPrPE)_2_(H_2_)H^+^ and trans-Ru(DMeOPrPE)_2_(H_2_)H^+^ by reaction of the trans-M(DMeOPrPE)_2_Cl_2_ complexes with H_2_ (Miller et al., 2002, Szymczak et al., 2006). In order to synthesize the analogous H_2_ complex of Os, we synthesized the trans-Os(DMeOPrPE)_2_Cl_2_ complex reported here.

The structure shows an octahedral coordination environment around osmium with trans-chloride ligands (Fig. 1).  Crystallization of the osmium congener allowed a direct comparison of trans-M(DMeOPrPE)_2_Cl_2_ complexes down the group 8 triad (Table 1).  The M-L bonds were found to increase substantially from iron to ruthenium with minimal elongation from ruthenium to osmium. This minimal change in going from ruthenium to osmium is consistent with a lanthanide contraction of the atomic radius.

### Experimental

To a flask containing [OsCl_6_][NEt_4_]_2_ (0.9114 g, 1.374 mmol) and NaBPh_4_ (1.890 g, 5.523 mmol) was added a solution of DMeOPrPE (2.11 g, 5.524 mmol) in methanol (300 ml) followed by ethanol (365 ml). After heating to reflux for 16 h, the solution was deep purple with a white precipitate, and the solution was kept at reflux. After 4 days, the solution was clear yellow with a white solid on the flask walls. The solvent was removed and the residue was extracted with hot hexanes (3 *x* 30 ml) and diethyl ether (3 *x* 30 ml), leaving a yellow oil. Upon letting the oil stand for 24 h, yellow crystals of *trans*-[Os(DMeOPrPE)_2_Cl_2_] developed. Yield: 0.862 g (61%); {^1^H} ^31^P NMR: d 8.6.

### Refinement

The structure was solved using direct methods and refined with anisotropic thermal parameters for non-H atoms. H atoms were positioned geometrically and refined in a rigid group model, C—H = 1.2*U*_eq_(C) and 1.5*U*_eq_(C), respectively for –CH_2_ and –CH_3_ groups. In the molecule there are eight -(CH_2_)_3_OCH_3_ terminal groups which are flexible and thermal parameters for atoms in these groups are significantly elongated. One of the –CH_2_OCH_3_ groups, C(17)O(4) C(18), is disordered over two positions in the ratio 0.700/0.300. Restrictions have been used in the refinement of this group; the typical values of the –CH_2_—CH_2_–, –CH_2_—O– and –O—CH_3_ bonds (1.524, 1.426 and 1.416 Å, respectively) have been used in the refinement as the targets for corresponding bond lengths.

### Crystal data

**Table:**

[OsCl_2_(C_18_H_40_O_4_P_2_)_2_]	*F*(000) = 1060
*M**_r_* = 1025.98	*D*_x_ = 1.459 Mg m^−^^3^
Monoclinic, *P*2_1_/*n*	Mo *K*α radiation, λ = 0.71073 Å
Hall symbol: -P 2yn	Cell parameters from 8415 reflections
*a* = 12.667 (3) Å	θ = 2.3–26.7°
*b* = 10.321 (2) Å	µ = 3.03 mm^−^^1^
*c* = 18.754 (4) Å	*T* = 173 K
β = 107.779 (3)°	Plate, yellow
*V* = 2335.0 (9) Å^3^	0.14 × 0.10 × 0.04 mm
*Z* = 2	

### Data collection

**Table:**

Bruker APEX CCD diffractometer	5333 independent reflections
Radiation source: fine-focus sealed tube	4423 reflections with *I* > 2σ(*I*)
graphite	*R*_int_ = 0.032
phi and ω scans	θ_max_ = 27.5°, θ_min_ = 1.7°
Absorption correction: multi-scan (*SADABS*; Sheldrick, 1995)	*h* = −16→16
*T*_min_ = 0.677, *T*_max_ = 0.889	*k* = −13→13
25950 measured reflections	*l* = −23→24

### Refinement

**Table:**

Refinement on *F*^2^	Primary atom site location: structure-invariant direct methods
Least-squares matrix: full	Secondary atom site location: difference Fourier map
*R*[*F*^2^ > 2σ(*F*^2^)] = 0.024	Hydrogen site location: inferred from neighbouring sites
*wR*(*F*^2^) = 0.058	H-atom parameters constrained
*S* = 1.04	*w* = 1/[σ^2^(*F*_o_^2^) + (0.028*P*)^2^ + 0.9782*P*] where *P* = (*F*_o_^2^ + 2*F*_c_^2^)/3
5333 reflections	(Δ/σ)_max_ < 0.001
251 parameters	Δρ_max_ = 0.87 e Å^−^^3^
5 restraints	Δρ_min_ = −0.51 e Å^−^^3^

### Special details

**Table:**

Geometry. All e.s.d.'s (except the e.s.d. in the dihedral angle between two l.s. planes)are estimated using the full covariance matrix. The cell e.s.d.'s are takeninto account individually in the estimation of e.s.d.'s in distances, anglesand torsion angles; correlations between e.s.d.'s in cell parameters are onlyused when they are defined by crystal symmetry. An approximate (isotropic)treatment of cell e.s.d.'s is used for estimating e.s.d.'s involving l.s. planes.
Refinement. Refinement of *F*^2^ against ALL reflections. The weighted *R*-factor *wR* and goodness of fit *S* are based on *F*^2^, conventional *R*-factors *R* are based on *F*, with *F* set to zero for negative *F*^2^. The threshold expression of *F*^2^ > σ(*F*^2^) is used only for calculating *R*-factors(gt) *etc*. and is not relevant to the choice of reflections for refinement. *R*-factors based on *F*^2^ are statistically about twice as large as those based on *F*, and *R*- factors based on ALL data will be even larger.

### Fractional atomic coordinates and isotropic or equivalent isotropic displacement parameters (Å^2^)

**Table:**

	*x*	*y*	*z*	*U*_iso_*/*U*_eq_	Occ. (<1)
Os1	0.5000	0.5000	0.0000	0.02110 (5)	
Cl1	0.31396 (6)	0.41077 (7)	−0.05728 (4)	0.03191 (16)	
P1	0.57375 (6)	0.32518 (7)	−0.04887 (4)	0.02556 (15)	
P2	0.53170 (6)	0.36384 (7)	0.10408 (4)	0.02567 (15)	
O1	0.9493 (2)	0.2799 (3)	−0.02252 (14)	0.0575 (7)	
O2	0.5043 (2)	0.1314 (3)	−0.27322 (14)	0.0666 (8)	
O3	0.5690 (2)	0.4496 (3)	0.35068 (13)	0.0512 (6)	
C1	0.5965 (3)	0.1882 (3)	0.01729 (16)	0.0354 (7)	
H1A	0.5270	0.1384	0.0089	0.043*	
H1B	0.6539	0.1295	0.0096	0.043*	
C2	0.6341 (3)	0.2421 (3)	0.09683 (16)	0.0352 (7)	
H2A	0.7082	0.2823	0.1076	0.042*	
H2B	0.6386	0.1715	0.1334	0.042*	
C3	0.7111 (2)	0.3487 (3)	−0.05918 (18)	0.0355 (7)	
H3A	0.7085	0.4293	−0.0883	0.043*	
H3B	0.7632	0.3645	−0.0086	0.043*	
C4	0.7620 (3)	0.2440 (3)	−0.09552 (18)	0.0397 (7)	
H4A	0.7657	0.1617	−0.0677	0.048*	
H4B	0.7141	0.2298	−0.1475	0.048*	
C5	0.8773 (3)	0.2810 (4)	−0.09625 (19)	0.0457 (8)	
H5A	0.9035	0.2188	−0.1273	0.055*	
H5B	0.8759	0.3685	−0.1182	0.055*	
C6	1.0568 (3)	0.3250 (5)	−0.0175 (2)	0.0691 (12)	
H6A	1.1037	0.3219	0.0348	0.104*	
H6B	1.0521	0.4144	−0.0358	0.104*	
H6C	1.0889	0.2699	−0.0481	0.104*	
C7	0.4913 (3)	0.2555 (3)	−0.13885 (17)	0.0336 (7)	
H7A	0.4127	0.2782	−0.1465	0.040*	
H7B	0.5140	0.2990	−0.1789	0.040*	
C8	0.4975 (3)	0.1093 (3)	−0.15003 (18)	0.0410 (8)	
H8A	0.5761	0.0816	−0.1339	0.049*	
H8B	0.4593	0.0638	−0.1185	0.049*	
C9	0.4444 (3)	0.0725 (3)	−0.23097 (19)	0.0455 (8)	
H9A	0.4455	−0.0228	−0.2367	0.055*	
H9B	0.3664	0.1021	−0.2481	0.055*	
C10	0.4635 (4)	0.1030 (6)	−0.3488 (2)	0.0944 (18)	
H10A	0.5089	0.1464	−0.3755	0.142*	
H10B	0.3867	0.1333	−0.3683	0.142*	
H10C	0.4659	0.0091	−0.3560	0.142*	
C11	0.5917 (2)	0.4287 (3)	0.19857 (16)	0.0303 (6)	
H11A	0.6554	0.4842	0.1984	0.036*	
H11B	0.5356	0.4853	0.2097	0.036*	
C12	0.6318 (3)	0.3317 (3)	0.26298 (17)	0.0381 (7)	
H12A	0.5718	0.2690	0.2608	0.046*	
H12B	0.6959	0.2829	0.2571	0.046*	
C13	0.6655 (3)	0.3987 (3)	0.33861 (18)	0.0437 (8)	
H13A	0.7016	0.3361	0.3786	0.052*	
H13B	0.7187	0.4693	0.3393	0.052*	
C14	0.5931 (5)	0.5225 (4)	0.4180 (3)	0.0833 (16)	
H14A	0.5240	0.5559	0.4241	0.125*	
H14B	0.6419	0.5950	0.4157	0.125*	
H14C	0.6301	0.4667	0.4606	0.125*	
C15	0.4218 (3)	0.2540 (3)	0.11326 (17)	0.0374 (7)	
H15A	0.3810	0.2211	0.0628	0.045*	
H15B	0.4577	0.1787	0.1438	0.045*	
C16	0.3385 (3)	0.3093 (3)	0.1475 (2)	0.0509 (9)	
H16A	0.2935	0.3763	0.1140	0.061*	
H16B	0.3777	0.3508	0.1960	0.061*	
C17	0.2609 (4)	0.2002 (6)	0.1602 (3)	0.0890 (18)	0.700 (6)
H17A	0.1975	0.2396	0.1727	0.107*	0.700 (6)
H17B	0.2313	0.1495	0.1136	0.107*	0.700 (6)
O4	0.3138 (3)	0.1245 (3)	0.2135 (2)	0.0515 (12)	0.700 (6)
C18	0.2400 (6)	0.0305 (5)	0.2282 (4)	0.0579 (17)	0.700 (6)
H18A	0.2813	−0.0264	0.2690	0.087*	0.700 (6)
H18B	0.2072	−0.0213	0.1830	0.087*	0.700 (6)
H18C	0.1812	0.0747	0.2426	0.087*	0.700 (6)
C17A	0.2609 (4)	0.2002 (6)	0.1602 (3)	0.0890 (18)	0.300 (6)
H17C	0.2140	0.1721	0.1101	0.107*	0.300 (6)
H17D	0.3090	0.1257	0.1825	0.107*	0.300 (6)
O4A	0.2061 (9)	0.2156 (9)	0.1940 (6)	0.072 (4)	0.300 (6)
C18A	0.1540 (15)	0.0978 (17)	0.2088 (10)	0.098 (7)	0.300 (6)
H18D	0.1048	0.1184	0.2387	0.146*	0.300 (6)
H18E	0.2112	0.0368	0.2364	0.146*	0.300 (6)
H18F	0.1106	0.0586	0.1612	0.146*	0.300 (6)

### Atomic displacement parameters (Å^2^)

**Table:**

	*U*^11^	*U*^22^	*U*^33^	*U*^12^	*U*^13^	*U*^23^
Os1	0.02079 (8)	0.01550 (8)	0.02816 (8)	−0.00026 (6)	0.00918 (6)	0.00023 (6)
Cl1	0.0249 (3)	0.0302 (4)	0.0410 (4)	−0.0056 (3)	0.0105 (3)	−0.0045 (3)
P1	0.0281 (4)	0.0187 (3)	0.0315 (4)	0.0028 (3)	0.0115 (3)	0.0000 (3)
P2	0.0300 (4)	0.0183 (3)	0.0300 (4)	0.0004 (3)	0.0112 (3)	0.0013 (3)
O1	0.0332 (13)	0.091 (2)	0.0499 (15)	0.0038 (14)	0.0149 (11)	0.0043 (14)
O2	0.080 (2)	0.078 (2)	0.0400 (14)	−0.0198 (16)	0.0150 (14)	−0.0137 (14)
O3	0.0617 (17)	0.0534 (14)	0.0407 (14)	−0.0105 (13)	0.0189 (12)	−0.0129 (12)
C1	0.0489 (19)	0.0217 (15)	0.0398 (17)	0.0087 (13)	0.0196 (15)	0.0028 (13)
C2	0.0464 (18)	0.0263 (15)	0.0345 (16)	0.0116 (13)	0.0146 (14)	0.0071 (12)
C3	0.0338 (16)	0.0309 (16)	0.0464 (18)	0.0029 (13)	0.0188 (14)	−0.0018 (14)
C4	0.0377 (18)	0.0457 (19)	0.0392 (18)	0.0094 (15)	0.0169 (15)	−0.0033 (15)
C5	0.0375 (18)	0.060 (2)	0.047 (2)	0.0101 (16)	0.0230 (16)	−0.0002 (17)
C6	0.036 (2)	0.097 (4)	0.074 (3)	0.004 (2)	0.016 (2)	−0.002 (3)
C7	0.0354 (17)	0.0254 (15)	0.0382 (17)	0.0027 (12)	0.0088 (14)	−0.0059 (13)
C8	0.057 (2)	0.0219 (16)	0.0427 (18)	−0.0017 (14)	0.0134 (16)	−0.0029 (13)
C9	0.051 (2)	0.0304 (18)	0.050 (2)	−0.0037 (15)	0.0078 (17)	−0.0116 (15)
C10	0.074 (3)	0.160 (6)	0.044 (3)	0.007 (3)	0.011 (2)	−0.018 (3)
C11	0.0334 (16)	0.0243 (16)	0.0323 (15)	0.0008 (12)	0.0086 (13)	0.0014 (12)
C12	0.0465 (19)	0.0299 (17)	0.0347 (16)	0.0052 (14)	0.0074 (14)	0.0044 (13)
C13	0.051 (2)	0.0391 (19)	0.0338 (17)	−0.0034 (16)	0.0019 (15)	0.0051 (14)
C14	0.115 (5)	0.076 (3)	0.061 (3)	−0.019 (3)	0.031 (3)	−0.033 (2)
C15	0.0468 (19)	0.0304 (17)	0.0363 (17)	−0.0084 (14)	0.0148 (15)	0.0023 (13)
C16	0.046 (2)	0.048 (2)	0.065 (2)	−0.0052 (17)	0.0256 (18)	0.0135 (18)
C17	0.080 (3)	0.128 (5)	0.076 (3)	0.005 (3)	0.048 (3)	0.044 (3)
O4	0.041 (2)	0.058 (2)	0.056 (2)	−0.0074 (17)	0.0166 (17)	0.0144 (18)
C18	0.059 (4)	0.047 (3)	0.080 (4)	−0.018 (3)	0.042 (4)	0.005 (3)
C17A	0.080 (3)	0.128 (5)	0.076 (3)	0.005 (3)	0.048 (3)	0.044 (3)
O4A	0.077 (7)	0.062 (6)	0.077 (7)	−0.007 (5)	0.024 (6)	0.012 (5)
C18A	0.076 (12)	0.099 (14)	0.105 (13)	−0.061 (11)	0.009 (10)	0.042 (11)

### Geometric parameters (Å, °)

**Table:**

Os1—P2	2.3383 (8)	C8—C9	1.509 (4)
Os1—P2^i^	2.3383 (8)	C8—H8A	0.9900
Os1—P1^i^	2.3434 (8)	C8—H8B	0.9900
Os1—P1	2.3434 (8)	C9—H9A	0.9900
Os1—Cl1	2.4515 (8)	C9—H9B	0.9900
Os1—Cl1^i^	2.4515 (8)	C10—H10A	0.9800
P1—C3	1.825 (3)	C10—H10B	0.9800
P1—C7	1.838 (3)	C10—H10C	0.9800
P1—C1	1.845 (3)	C11—C12	1.531 (4)
P2—C11	1.829 (3)	C11—H11A	0.9900
P2—C2	1.840 (3)	C11—H11B	0.9900
P2—C15	1.844 (3)	C12—C13	1.517 (4)
O1—C5	1.405 (4)	C12—H12A	0.9900
O1—C6	1.414 (4)	C12—H12B	0.9900
O2—C10	1.384 (5)	C13—H13A	0.9900
O2—C9	1.392 (4)	C13—H13B	0.9900
O3—C13	1.412 (4)	C14—H14A	0.9800
O3—C14	1.420 (5)	C14—H14B	0.9800
C1—C2	1.525 (4)	C14—H14C	0.9800
C1—H1A	0.9900	C15—C16	1.506 (5)
C1—H1B	0.9900	C15—H15A	0.9900
C2—H2A	0.9900	C15—H15B	0.9900
C2—H2B	0.9900	C16—C17	1.560 (6)
C3—C4	1.521 (4)	C16—H16A	0.9900
C3—H3A	0.9900	C16—H16B	0.9900
C3—H3B	0.9900	C17—O4	1.283 (5)
C4—C5	1.514 (4)	C17—H17A	0.9900
C4—H4A	0.9900	C17—H17B	0.9900
C4—H4B	0.9900	O4—C18	1.431 (6)
C5—H5A	0.9900	C18—H18A	0.9800
C5—H5B	0.9900	C18—H18B	0.9800
C6—H6A	0.9800	C18—H18C	0.9800
C6—H6B	0.9800	O4A—C18A	1.451 (13)
C6—H6C	0.9800	C18A—H18D	0.9800
C7—C8	1.528 (4)	C18A—H18E	0.9800
C7—H7A	0.9900	C18A—H18F	0.9800
C7—H7B	0.9900		
			
P2—Os1—P2^i^	180.00 (4)	C9—C8—H8A	109.4
P2—Os1—P1^i^	97.10 (3)	C7—C8—H8A	109.4
P2^i^—Os1—P1^i^	82.90 (3)	C9—C8—H8B	109.4
P2—Os1—P1	82.90 (3)	C7—C8—H8B	109.4
P2^i^—Os1—P1	97.10 (3)	H8A—C8—H8B	108.0
P1^i^—Os1—P1	180.00 (3)	O2—C9—C8	108.1 (3)
P2—Os1—Cl1	92.13 (3)	O2—C9—H9A	110.1
P2^i^—Os1—Cl1	87.87 (3)	C8—C9—H9A	110.1
P1^i^—Os1—Cl1	90.91 (3)	O2—C9—H9B	110.1
P1—Os1—Cl1	89.09 (3)	C8—C9—H9B	110.1
P2—Os1—Cl1^i^	87.87 (3)	H9A—C9—H9B	108.4
P2^i^—Os1—Cl1^i^	92.13 (3)	O2—C10—H10A	109.5
P1^i^—Os1—Cl1^i^	89.09 (3)	O2—C10—H10B	109.5
P1—Os1—Cl1^i^	90.91 (3)	H10A—C10—H10B	109.5
Cl1—Os1—Cl1^i^	180.0	O2—C10—H10C	109.5
C3—P1—C7	104.32 (15)	H10A—C10—H10C	109.5
C3—P1—C1	102.40 (15)	H10B—C10—H10C	109.5
C7—P1—C1	104.24 (14)	C12—C11—P2	117.7 (2)
C3—P1—Os1	116.50 (10)	C12—C11—H11A	107.9
C7—P1—Os1	118.55 (10)	P2—C11—H11A	107.9
C1—P1—Os1	109.03 (10)	C12—C11—H11B	107.9
C11—P2—C2	103.16 (14)	P2—C11—H11B	107.9
C11—P2—C15	103.69 (14)	H11A—C11—H11B	107.2
C2—P2—C15	98.98 (15)	C13—C12—C11	111.8 (2)
C11—P2—Os1	120.25 (10)	C13—C12—H12A	109.3
C2—P2—Os1	107.11 (10)	C11—C12—H12A	109.3
C15—P2—Os1	120.36 (11)	C13—C12—H12B	109.3
C5—O1—C6	112.7 (3)	C11—C12—H12B	109.3
C10—O2—C9	113.1 (3)	H12A—C12—H12B	107.9
C13—O3—C14	112.2 (3)	O3—C13—C12	108.0 (3)
C2—C1—P1	108.5 (2)	O3—C13—H13A	110.1
C2—C1—H1A	110.0	C12—C13—H13A	110.1
P1—C1—H1A	110.0	O3—C13—H13B	110.1
C2—C1—H1B	110.0	C12—C13—H13B	110.1
P1—C1—H1B	110.0	H13A—C13—H13B	108.4
H1A—C1—H1B	108.4	O3—C14—H14A	109.5
C1—C2—P2	107.7 (2)	O3—C14—H14B	109.5
C1—C2—H2A	110.2	H14A—C14—H14B	109.5
P2—C2—H2A	110.2	O3—C14—H14C	109.5
C1—C2—H2B	110.2	H14A—C14—H14C	109.5
P2—C2—H2B	110.2	H14B—C14—H14C	109.5
H2A—C2—H2B	108.5	C16—C15—P2	117.0 (2)
C4—C3—P1	120.1 (2)	C16—C15—H15A	108.0
C4—C3—H3A	107.3	P2—C15—H15A	108.0
P1—C3—H3A	107.3	C16—C15—H15B	108.0
C4—C3—H3B	107.3	P2—C15—H15B	108.0
P1—C3—H3B	107.3	H15A—C15—H15B	107.3
H3A—C3—H3B	106.9	C15—C16—C17	110.3 (3)
C5—C4—C3	111.5 (3)	C15—C16—H16A	109.6
C5—C4—H4A	109.3	C17—C16—H16A	109.6
C3—C4—H4A	109.3	C15—C16—H16B	109.6
C5—C4—H4B	109.3	C17—C16—H16B	109.6
C3—C4—H4B	109.3	H16A—C16—H16B	108.1
H4A—C4—H4B	108.0	O4—C17—C16	110.8 (4)
O1—C5—C4	109.1 (3)	O4—C17—H17A	109.5
O1—C5—H5A	109.9	C16—C17—H17A	109.5
C4—C5—H5A	109.9	O4—C17—H17B	109.5
O1—C5—H5B	109.9	C16—C17—H17B	109.5
C4—C5—H5B	109.9	H17A—C17—H17B	108.1
H5A—C5—H5B	108.3	C17—O4—C18	110.3 (4)
O1—C6—H6A	109.5	O4—C18—H18A	109.5
O1—C6—H6B	109.5	O4—C18—H18B	109.5
H6A—C6—H6B	109.5	H18A—C18—H18B	109.5
O1—C6—H6C	109.5	O4—C18—H18C	109.5
H6A—C6—H6C	109.5	H18A—C18—H18C	109.5
H6B—C6—H6C	109.5	H18B—C18—H18C	109.5
C8—C7—P1	118.3 (2)	O4A—C18A—H18D	109.5
C8—C7—H7A	107.7	O4A—C18A—H18E	109.5
P1—C7—H7A	107.7	H18D—C18A—H18E	109.5
C8—C7—H7B	107.7	O4A—C18A—H18F	109.5
P1—C7—H7B	107.7	H18D—C18A—H18F	109.5
H7A—C7—H7B	107.1	H18E—C18A—H18F	109.5
C9—C8—C7	111.2 (3)		
			
P2—Os1—P1—C3	−109.55 (12)	C7—P1—C1—C2	−163.7 (2)
P2^i^—Os1—P1—C3	70.45 (12)	Os1—P1—C1—C2	−36.2 (2)
P1^i^—Os1—P1—C3	5(47)	P1—C1—C2—P2	53.3 (2)
Cl1—Os1—P1—C3	158.19 (12)	C11—P2—C2—C1	−175.8 (2)
Cl1^i^—Os1—P1—C3	−21.81 (12)	C15—P2—C2—C1	77.8 (2)
P2—Os1—P1—C7	124.59 (12)	Os1—P2—C2—C1	−47.9 (2)
P2^i^—Os1—P1—C7	−55.41 (12)	C7—P1—C3—C4	−43.0 (3)
P1^i^—Os1—P1—C7	−121 (47)	C1—P1—C3—C4	65.5 (3)
Cl1—Os1—P1—C7	32.34 (12)	Os1—P1—C3—C4	−175.7 (2)
Cl1^i^—Os1—P1—C7	−147.66 (12)	P1—C3—C4—C5	−178.2 (2)
P2—Os1—P1—C1	5.65 (11)	C6—O1—C5—C4	−174.6 (3)
P2^i^—Os1—P1—C1	−174.35 (11)	C3—C4—C5—O1	68.8 (4)
P1^i^—Os1—P1—C1	120 (47)	C3—P1—C7—C8	83.9 (3)
Cl1—Os1—P1—C1	−86.61 (11)	C1—P1—C7—C8	−23.2 (3)
Cl1^i^—Os1—P1—C1	93.39 (11)	Os1—P1—C7—C8	−144.6 (2)
P2^i^—Os1—P2—C11	88 (100)	P1—C7—C8—C9	−168.4 (2)
P1^i^—Os1—P2—C11	−43.13 (12)	C10—O2—C9—C8	179.3 (4)
P1—Os1—P2—C11	136.87 (12)	C7—C8—C9—O2	62.8 (4)
Cl1—Os1—P2—C11	−134.31 (12)	C2—P2—C11—C12	−49.0 (3)
Cl1^i^—Os1—P2—C11	45.69 (12)	C15—P2—C11—C12	53.8 (3)
P2^i^—Os1—P2—C2	−29 (100)	Os1—P2—C11—C12	−168.09 (19)
P1^i^—Os1—P2—C2	−160.21 (11)	P2—C11—C12—C13	−172.6 (2)
P1—Os1—P2—C2	19.79 (11)	C14—O3—C13—C12	−175.8 (3)
Cl1—Os1—P2—C2	108.61 (11)	C11—C12—C13—O3	68.3 (3)
Cl1^i^—Os1—P2—C2	−71.39 (11)	C11—P2—C15—C16	52.9 (3)
P2^i^—Os1—P2—C15	−141 (100)	C2—P2—C15—C16	158.9 (3)
P1^i^—Os1—P2—C15	88.10 (12)	Os1—P2—C15—C16	−85.1 (3)
P1—Os1—P2—C15	−91.90 (12)	P2—C15—C16—C17	−173.0 (3)
Cl1—Os1—P2—C15	−3.08 (12)	C15—C16—C17—O4	70.8 (5)
Cl1^i^—Os1—P2—C15	176.92 (12)	C16—C17—O4—C18	175.9 (4)
C3—P1—C1—C2	87.8 (2)		

Symmetry codes: (i) −*x*+1, −*y*+1, −*z*.
